# Effect of Different-Volume Fluid Resuscitation on Organ Functions in Severe Acute Pancreatitis and Therapeutic Effect of *Poria cocos*

**DOI:** 10.1155/2020/6408202

**Published:** 2020-10-14

**Authors:** Xiao-Lin Yi, Jing Hu, Qiu-Ting Wu, Yu-Mei Zhang, Qian Hu, Ling Yuan, Yi-Fan Miao, Huan Chen, Lv Zhu, Juan Li, Xian-Lin Zhao, Jia-Qi Yao, Xiao-Yu Dai, Mei-Hua Wan, Wen-Fu Tang

**Affiliations:** ^1^Department of Integrated Traditional Chinese and Western Medicine, West China Hospital, Sichuan University, Chengdu 610041, China; ^2^Department of Gastroenterology, Leshan Municipal Hospital of Traditional Chinese Medicine, Leshan 614000, China; ^3^Department of Traditional Chinese Medicine, Xiang'an Hospital of Xiamen University, Xiamen 361101, China

## Abstract

**Objective:**

To explore the effect of different-volume fluid resuscitation (FR) on organ functions in severe acute pancreatitis (SAP) and to elucidate the therapeutic effect and mechanism of *Poria cocos* on organ injuries caused by high-volume FR.

**Methods:**

1. Clinical study: retrospective analysis of thirty-one patients about the effect of titrated fluid resuscitation protocol (TFR) on the occurrence of acute kidney injury (AKI) secondary to SAP. 2. Experimental study: rats (*N* = 30) were randomly divided into five groups: sham, model, low-volume FR (1.5 ml/kg/h), high-volume FR (10 ml/kg/h), and *Poria cocos* combined with high-volume FR (10 ml/kg/h + intraintestinal administration *Poria cocos* 5 g/kg); serum or plasma indicators and histopathologic scores were compared to explore the effect and mechanism of different fluid volumes and *Poria cocos* on organ function in SAP.

**Results:**

The occurrence of AKI, fluid volume, and fluid velocity in TFR group was lower than that in the control group. Logistic regression analysis showed that increased Marshall scores and fluid velocity were risk factors for predicting occurrence of AKI in SAP. Low-volume FR decreased the levels of blood urea nitrogen (BUN), serum creatinine (Cr), matrix metalloproteinase (MMP), and pathologic scores of the pancreas and kidney. High-volume FR increased ascites, MMPs, and kidney pathologic scores. *Poria cocos* decreased the levels of BUN, Cr, MMPs, and pathologic scores of the pancreas and kidney and increased the arterial oxygen saturation.

**Conclusion:**

TFR-associated lower fluid volume and velocity reduced the occurrence of AKI secondary to SAP. High volume might aggravate AKI via increased MMP release leading to endothelial glycocalyx damage and vascular endothelial dysfunction. *Poria cocos* reduced MMP release, relieved glycocalyx damage, and alleviated the pancreas and kidney injury aggravated by high fluid volume in SAP. Therefore, endothelial glycocalyx protection might be a new strategy in the treatment of SAP.

## 1. Introduction

Acute pancreatitis (AP) is a common acute inflammatory disease in pancreas, and majority of this disease is mild and self-limited. The number of people attacked by AP goes up worldwide and 10%–20% of the mild cases progress to severe acute pancreatitis (SAP) with a mortality of 10% to 30% among those severe cases [1, 2]. AP patients with persistent organ failure are diagnosed as SAP, and kidney is one of the most common organs involved [3]. The incidence of acute kidney injury (AKI) secondary to SAP ranges from 15.05% to 69.3% [4, 5]. Mortality of SAP companied with AKI rises by one to three times than that of SAP without AKI [5, 6].

Vascular leakage induced by inflammatory exudation increased the risk of hypovolemia, organ hypoperfusion, and extrapancreatic organ injuries like the kidney [7]. Early fluid resuscitation can restore the intravascular volume, ameliorate the organ perfusion, and reduce the mortality and is recommended as the basic treatment of SAP [8, 9]. High-volume fluid resuscitation can rapidly restore the intravascular volume but involves positive cumulative fluid balance which may deteriorate respiratory and kidney injuries in AP [10, 11]. However, the mechanism of high volume-related kidney injury remains elusive. Early goal-directed fluid therapy can decrease excessive positive fluid balance and mortality and benefit more to patients than high volume [12, 13]. Current guidelines recommend goal-directed fluid resuscitation in early phase management of SAP, but optimal protocol including topics of fluid volume, velocity and initial time is debated [14, 15].

Why does organ dysfunction still exist after partial or complete fluid resuscitation? Microcirculation dysfunction contributed to multiple complications in SAP [16]. Fluid resuscitation improves tissue perfusion but microcirculation dysfunction induced by inflammation or heterogeneous perfusion recovery still exists [17]. Vascular endothelial barrier dysfunction plays a key role in microcirculation dysfunction. Endothelial glycocalyx is an important component of vascular endothelium skeleton and greatly affects endothelial integrity and endothelial barrier stabilization, especially in vascular permeability regulation [18, 19]. Syndecan-1, heparin sulfate, and hyaluronan are major components of glycocalyx and critical biomarkers for assessing glycocalyx damage [18, 19]. Matrix metalloproteinases (MMPs), an important cutter of endothelial glycocalyx, causes glycocalyx degradation, and MMP inhibition can significantly reduce glycocalyx injury and vascular leakage [20]. Glycocalyx degradation-related microcirculation disturbance exists in sepsis and acute pancreatitis [19, 21]. The mechanism of organ injury caused by high-volume fluid resuscitation in the treatment of hypovolemia and organ hypoperfusion in SAP remains unclear. It is not known whether endothelial glycocalyx degradation involves high volume-related organ injuries in SAP. *Poria cocos*, a traditional Chinese medicine, has the functions of promoting water and dampness distribution and metabolism and promoting the spleen to metabolize water and dampness. Whether *Poria cocos* can alleviate organ dysfunction induced by high-volume fluid resuscitation needs further studies.

Little randomized controlled trials focused on AKI secondary to SAP. This study retrospectively analyzed the effect of different volume especially in titrated fluid resuscitation protocol-based volume on the occurrence of AKI among SAP patients. Furthermore, we designed an experiment to explore the effect and mechanism of different-volume fluid resuscitation on organ injuries and to elucidate the therapeutic effect and mechanism of *Poria cocos* on organ injuries caused by high-volume fluid resuscitation.

## 2. Methods

### 2.1. Clinical Study

#### 2.1.1. Design and Study Population

**Figure 1:**
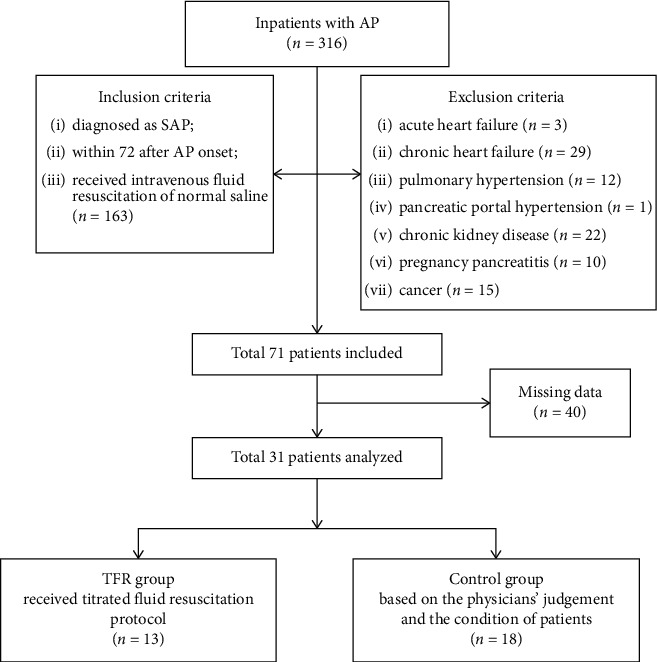
Flow diagram. AP: acute pancreatitis; SAP: severe acute pancreatitis; TFR: titrated fluid resuscitation protocol.

A retrospective analysis of the effect of the initial titrated fluid resuscitation (TFR) on the occurrence of AKI in patients with SAP was conducted at the West China Hospital of Sichuan University (Figure 1). Based on the Declaration of Helsinki, this trial was approved by Ethics Committee of West China Hospital, Sichuan University, and registered in ClinicalTrials.gov (Clinical Trials. Gov., identifiers: ChiCTR1800019855). SAP patients (≥18 years of age) admitted to hospital from June 2016 to May 2017 were included, and information was extracted from the electric medical record system including demographics, infusion volume, physiochemical measurements, complications, in-hospital death, and comorbidity. A total of 316 patients were identified, and 31 patients (TFR group 13 vs. the control group 18) were ultimately analyzed.

*(1) Inclusion Criteria*. (a) Diagnosed as SAP based on the revised Atlanta classification [3]; (b) admitted within 72 hours after AP symptoms onset; and (c) received intravenous fluid therapy of normal saline during hospitalization.

*(2) Exclusion Criteria*. (a) Acute or chronic heart failure; (b) pulmonary hypertension and pancreatic portal hypertension; (c) chronic kidney disease; (d) pregnancy pancreatitis; and (e) cancer.

#### 2.1.2. Interventions

Eligible patients were divided into the TFR group and control group. The TFR group received titrated fluid resuscitation (TFR) protocols established according to guidelines and previous study: (i) when three of the following five items met, fluid resuscitation was initiated at a rate of 3 ml/kg/h: (a) urinary output ≤ 0.5 ml/kg/h; (b) mean arterial pressure ≤ 65 mmHg; (c) heart rate ≥ 120 beats/min; (d) hematocrit ≥ 44%; and (e) blood lactic acid ≥ 4 mmol/L [8, 9, 11]. And (ii) when two of the following four items met after being monitored every 4–6 hours, fluid velocity was adjusted to 1.5 ml/kg/h: (a) urinary output ≥ 1 ml/kg/h; (b) heart rate < 120 beats/min; (c) hematocrit < 44%; (d) mean arterial pressure ≥ 65 mmHg [8, 9].

In the control group, the start-up of fluid resuscitation and the adjustment of fluid volume and velocity were based on the physicians' judgment according to the condition of patients.

Except for the different fluid resuscitation protocol, two-group patients appropriately received the routine management including analgesic, decompression, and nutrition based on the guidelines of AP [8].

#### 2.1.3. Measurement

*(1) Primary Outcomes*. The primary outcome was the occurrence of AKI within 72 h after admission. AKI was diagnosed if one of the following two items met: (a) serum creatinine ≥ 1.5 times of baseline or ≥ 26.5 *µ*mol/L; (b) urine less than 0.5 ml/kg/h lasted ≥ 6 consecutive hours [22]. Evaluation of AKI secondary to AP was also based on the renal injury score of Marshall scores in Revised Atlanta criteria [3, 23].

*(2) Secondary Outcomes*. The secondary outcomes were organ failure, death, length of stay, fluid input, and output condition within the first 72 h of admission (including intravenous fluid volume, velocity, initial time, and urine output), intensive care unit (ICU) admission, ventilator use, pancreatitis complications, pancreatitis-related surgery, and severity of AP. Patients' demographics consisted of age, gender, etiology, and body mass index (BMI).

Organ failure was diagnosed when one of three systems including respiratory, cardiovascular, or renal system scored 2 or more [3]. Organ failure lasting more than 48 h was diagnosed as persistent organ failure (POF). Marshall scores, acute physiology, Age and Chronic Health Evaluation II (APACHE-II) scores, and SIRS scores at admission were used to evaluate organ failure.

Fluid input and output conditions within the first 72 h of admission included the records of 24-hour input volume of intravenous fluid, 24-hour total input or output volume, and hourly urine output and intravenous fluid velocity to evaluate the responsiveness of fluid therapy. Heart rate, hematocrit, mean arterial pressure, and blood lactic acid were used to evaluate the responsiveness of fluid therapy and prognosis as well [8, 14].

Computed tomography (CT) scan findings of pneumonia, pericardial effusion, pleural effusion, and pancreatitis complications including peripancreatic fluid collection, pseudocyst, acute necrotic collection, and walled-off necrosis were recorded for the prediction of organ failure. CT scores and grades were calculated based on the modified computed tomography severity index (MCTSI) score and the Balthazar CT grade [24, 25]. Additionally, the serum level of total bilirubin, platelet, total cholesterol, and blood glucose were recorded to reflect the organ function and evaluate the existence of hepatic dysfunction, coagulation abnormality, hyperlipidaemia, or hyperglycemia [23].

### 2.2. Experimental Study

#### 2.2.1. Reagents and Materials

*Poria cocos* boil-free granules were prepared and purchased from the Affiliated Hospital of Chengdu University of Traditional Chinese Medicine (Chengdu, Sichuan Province, China). Pentobarbital sodium salt and sodium taurocholate were purchased from Chengdu Baoxin Biotechnology Co., Ltd (Chengdu, Sichuan Province, China) and Beijing bailingwei Technology Co., Ltd (Beijing, China) respectively. The Rat Syndecan-1 ELISA kit, SD MMP ELISA kit, SD hyaluronan ELISA kit, and SD soluble CD44 ELISA kit were purchased from Shanghai Chenyi Industrial Co., Ltd (Shanghai, China). *Y*-type and *Z*-type disposable intravenous indwelling needles (24 G) were purchased from Jiangxi Sanxin Medical Technology Co., Ltd (Jiangxi, China) and Henan Xinwei Medical Equipment Co., Ltd (Henan, China), respectively. The BL-420S biological function experimental system and PT-102 blood pressure sensor were provided by Chengdu Taimeng Technology Co., Ltd (Chengdu, China). Medical microtube (NO. #BB31695-PE/3, #BB518-20) were purchased from Scientific Commodities, Inc. (Lake Havasu, Arizona, USA).

#### 2.2.2. Animals

This animal experiment performed abiding by legislation on the Care and Use of Experimental Animal was approved by the Animal Ethical and Welfare Committee of West China Hospital, Sichuan University (No. 2019175A), and the results were reported according to the ARRIVE Guidelines (ARRIVE Checklist). 30 male Sprague-Dawley rats (body weight: 320 ± 30 g) were purchased from Experimental Animal Center of Sichuan University (Chengdu, China). Animals were raised adaptively in cages (5 rats per cage) with free diet for one week in independent ventilation system at 21 ± 1°C room temperature and 12 h light/dark cycle.

#### 2.2.3. Rat AP Modeling and Treatment

Thirty rats were randomly divided into five groups (*n* = 6 per group): sham group (SHAM), model group (NFR), low-volume fluid resuscitation group (LFR), high-volume fluid resuscitation group (HFR), and *Poria cocos* combined high-volume fluid resuscitation group (FL + HFR). All rats were fasted for 12 hours and drank freely before operation. Rats were anaesthetized by 2% pentobarbital sodium solution (50 mg/kg, i.p.), and one-fifth of the initial dose was administered intravenously per hour to maintain anesthesia. Then, rats were detained and heparinized (50 IU/mL) (vein needle at tail vein) (*Z*-type, 24 G) to establish infusion channel and left femoral artery (*Y*-type, 24 G) to continuously record mean arterial pressure and heart rate by the BL-420S biological function experimental system [26]. At 30 minutes after needle indwelling, rats were performed laparotomy in the sham group and 3.5% sodium taurocholate (1 mL/kg) retrograde cholangiopancreatic injection by the microinjection pump at the rate of 6 mL/h to establish AP model in the other four groups [27]. Rats in the FL + HFR group detained a medical microtube through the duodenal wall incision of the opposite of major duodenal papilla to establish the drug administration channel.

At 30 minutes after modeling, rats in the LFR (1.5 mL/kg/h), HFR (10 mL/kg/h), and FL + HFR (10 mL/kg/h) groups started the eight-hour fluid resuscitation of normal saline with the microinjection pump though the tail vein. *Poria cocos* boil-free granules were dissolved in distilled water with ultrasonic vortex dissolution for 15 minutes and heated in 37°C constant temperature water bath for 30 min before use. At two hours after modeling, rats in the FL + HFR were treated with the *Poria cocos* solution (1 g/mL) at the dose of 5 g/kg through the duodenal drug administration catheter. Next, all rats were conducted a two-hour observation and received euthanasia by overdose anesthesia finally.

#### 2.2.4. Blood Sample Analysis

At ten hours after fluid resuscitation start-up, all rats received euthanasia by overdose anesthesia. Blood sample were collected from the heart, centrifuged at 3000 rpm for 5 min at 21°C, and stored at −80°C. Serum examination of amylase, creatinine, blood urea nitrogen, and lactate were analyzed by the automatic biochemical analyzer (7170A, HITACHI, Tokyo, Japan) at the Chengdu Office Hospital of the people's Government of Tibet Autonomous Region. Plasma levels of Syndecan-1, matrix metalloproteinase (MMPs), soluble CD44, and serum hyaluronan were tested by enzyme-linked immunosorbent assay (ELISA) using ELISA kits according to product instruction. Arterial blood gas analysis was conducted by the automatic analyzer (ABL800, FLEX, Denmark, Radiometer Medical ApS).

#### 2.2.5. Ascites and Urine Output

Ascites was collected with a syringe after blood collection. Urine was collected by using a tube with a tape fixed to the external urethral orifice and measured by a syringe.

#### 2.2.6. Histopathological Analysis

Fresh tissue of the pancreas and kidney was fixed in 4% paraformaldehyde, dehydrated in gradient ethanol, embedded in paraffin, sectioned at a thickness 5 *μ*m, and performed with hematoxylin and eosin (H&E) staining. Stained slicers were observed and scored by two independent pathologists blinded to grouping according to histopathological scoring criteria [28, 29].

### 2.3. Statistical Analysis

Data were presented as mean ± standard deviation and median (interquartile: p25–p75) for normally and nonnormally distributed quantitative data, respectively. Categorical data were presented as frequency and percentage. Results were analyzed with PEMS 3.1 for Windows (Sichuan University, China) and reviewed by Dr. Hai Niu who came from College of Mathematics, Sichuan University. Two-group comparison was analyzed with Fisher's exact probability for categorical data and the Mann–Whitney *U* or Student's *t* test for nonnormally or normally distributed quantitative data, respectively. The multiple group comparison of normally distributed data was performed by one-way ANOVA with Bonferroni test for data in variance homogeneity and by the Welch test with ther Tamhane test for data in variance inhomogeneity. The same methods were used for repeated measurement data of mean arterial pressure and heart rate. The multiple-group comparison of nonnormally distributed data was performed by the KruskalWallis *H* test. Logistic and multiple line regression analyses were performed to assess impact factors of AKI. A two-sided *p* value < 0.05 was the statistical difference. Means replace the missing values.

## 3. Results

### 3.1. Clinical Study

#### 3.1.1. Baseline Characteristics

Thirty-one patients (13 in the TFR group and 18 in the control group) were included in the analysis (Figure 1). No statistical difference in age, gender, etiology, body mass index, onset time, SIRS score, APACHE-II score, CT scores, creatinine at admission, start-up, and duration of fluid resuscitation between two groups showed that the following differences were irrespective of baseline difference of two groups.

#### 3.1.2. TFR Associated with Lower Fluid Volume Reduced the Incidence of AKI

The TFR group had a lower median serum creatinine (67.7, 61.7–120.7 vs. 97.8, 72.6–152.3 *μ*mol/L, *p*=0.029) and lower incidence of AKI (2/13 vs. 10/18, *p*=0.032) than those in the control group.

#### 3.1.3. TFR Associated with Lower Fluid Volume Reduced the Early Organ Damage

At first 24 h after admission, the TFR group had lower organ failure (2/13 vs. 11/18, *p*=0.025), Marshall score (0.6 ± 1.0 vs. 2.2 ± 1.5, *p*=0.004), serum bilirubin (13.1, 9.4–23.3 vs. 26.5, 15.3–41.3 *μ*mol/L, *p*=0.018), and higher oxygenation index (431.0 ± 167.1 vs. 316.0 ± 118.8, *p*=0.034) than in the control group. As for use of mechanical ventilation, the TFR group started later (36 ± 28.4 vs. 10 ± 18.5 h, *p*=0.014) with shorter duration (88.5, 62.5–145.8 vs. 179, 158.3–270.8 h, *p*=0.016) than that of the control group. No significant difference was found in the occurrence of POF, persistent multiple organ failure, length of stay, and mortality.

#### 3.1.4. TFR Associated with Smaller Fluid Volume and Velocity

SAP patients in the TFR group received lower fluid volume (2875.0 ± 862.5 vs. 3515.0 ± 707.2 ml, *p*=0.002) and fluid velocity (158.0 ± 67.4 vs. 187.0 ± 45.9 ml/h, *p*=0.03) than those in the control group. The urine output of the TFR group in the first three days after admission was significantly lower than that of the control group: 24 h urine volume (721.0 ± 692.6 vs. 1149.0 ± 590.0, *p*=0.034), 48 h urine volume (1520.0 ± 663.4 vs. 3231.0 ± 1667.2, *p* < 0.001), and 72 h urine volume (2269.0 ± 1106.4 vs. 3186.0 ± 1214.9, *p*=0.047). No significant differences was found between the two groups in hourly urinary output, interval time between admission and fluid resuscitation, duration of fluid resuscitation, and total volumes of acetated Ringer's sodium and lactated Ringer's solution.

#### 3.1.5. TFR Improved the Prognosis of SAP

The TFR group had a shorter parenteral nutrition duration (120, 88–192 vs. 203, 152–247, *p*=0.017). Hematocrit is related to severity and mortality rate of AP. The hematocrit level of the TFR group reached to the goal (35%–44%) of fluid therapy in the first 48 h (0.44, 0.38–0.47 vs. 0.46, 0.38–0.48, *p*=0.007) and 72 h (0.42, 0.40–0.45 vs. 0.39, 0.34–0.40, *p*=0.015). There were no significant differences between two groups in the use of antibiotic, comorbidity, pancreatic complications, pancreatitis-related surgery, and ICU admission.

#### 3.1.6. Increased Fluid Velocity Increased the Risk of AKI Secondary to SAP

**Figure 2:**
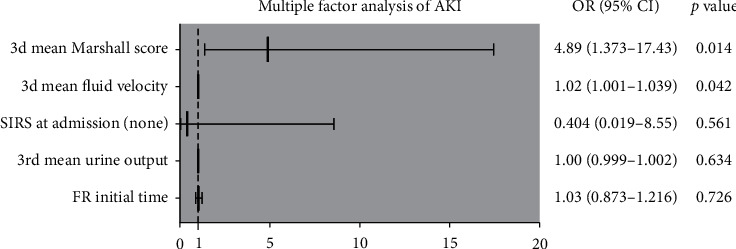
Multiple factor analysis of AKI. 3d: three days; SIRS: systemic inflammatory response syndrome; FR: fluid resuscitation; AKI: acute kidney injury.

To further analyze the impact of intravenous fluid on the occurrence of AKI, logistic regression analysis was applied and it showed that increased fluid velocity (OR = 1.02, 95% CI: 1.001–1.039, *p*=0.042) and Marshall score (OR = 4.893, 95% CI: 1.373–17.43, *p*=0.014) were risk factors for predicting AKI secondary to SAP (Figure 2). Then, we investigated the effect of different fluid velocities and volume on AKI in the SAP model.

### 3.2. Experimental Study

#### 3.2.1. Effect of Different-Volume Fluid Resuscitation on Organ Function

**Figure 3:**
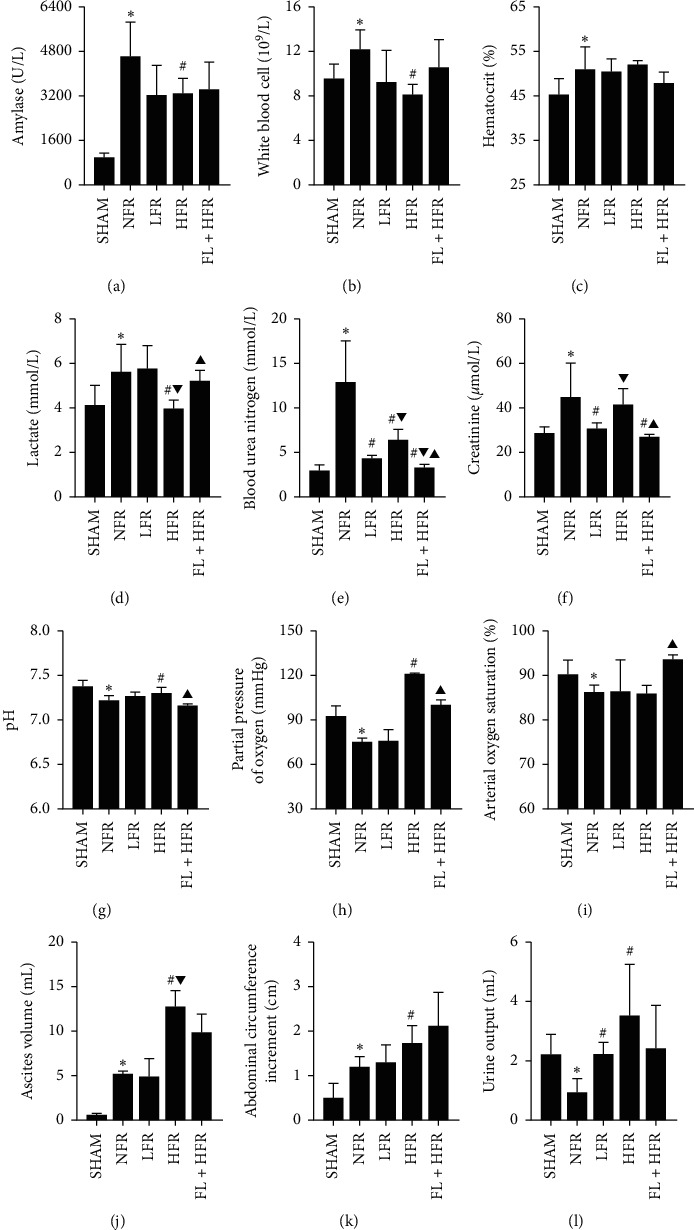
Comparison of organ injury in hematologic and biological indexes. AP: acute pancreatitis; SAP: severe acute pancreatitis; TFR: titrated fluid resuscitation protocol. SHAM: sham; NFR: nonfluid resuscitation; LFR: low-volume fluid resuscitation; HFR: high-volume fluid resuscitation; FL + HFR: *Poria cocos* combined with high-volume fluid resuscitation. ^∗, #, ▼, ▲^Compared with SHAM, NFR, LFR, and HFR groups respectively, *p* < 0.05.

**Figure 4:**
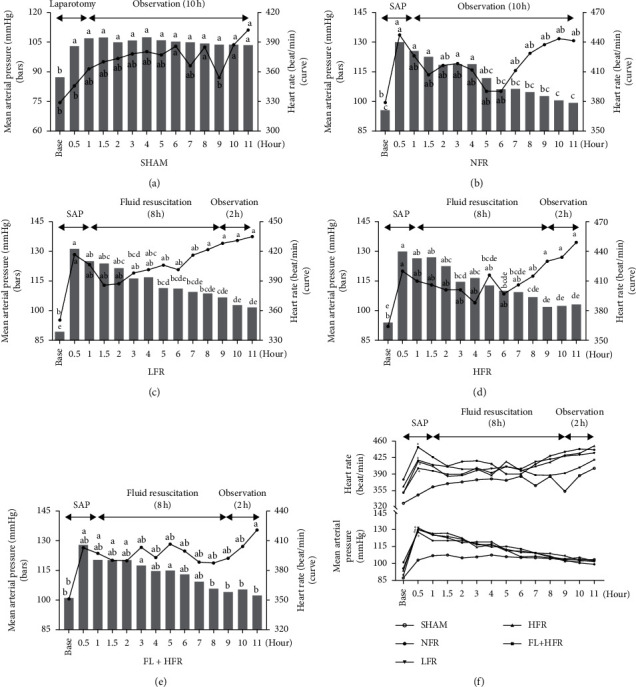
Comparison of mean arterial pressure and heart between five groups. SAP: severe acute pancreatitis; SHAM: sham; NFR: nonfluid resuscitation; LFR: low-volume fluid resuscitation; HFR: high-volume fluid resuscitation; FL + HFR: *Poria cocos* combined with high-volume fluid resuscitation. (a–e) statistical difference in alphabetic notation to show the difference in the same group at different time points. If there is no same letter, the difference is statistically significant. Black (curve): heart rate, gray (bar): mean arterial pressure; ^*∗*^Compared with the SHAM group, *p* < 0.05.

**Figure 5:**
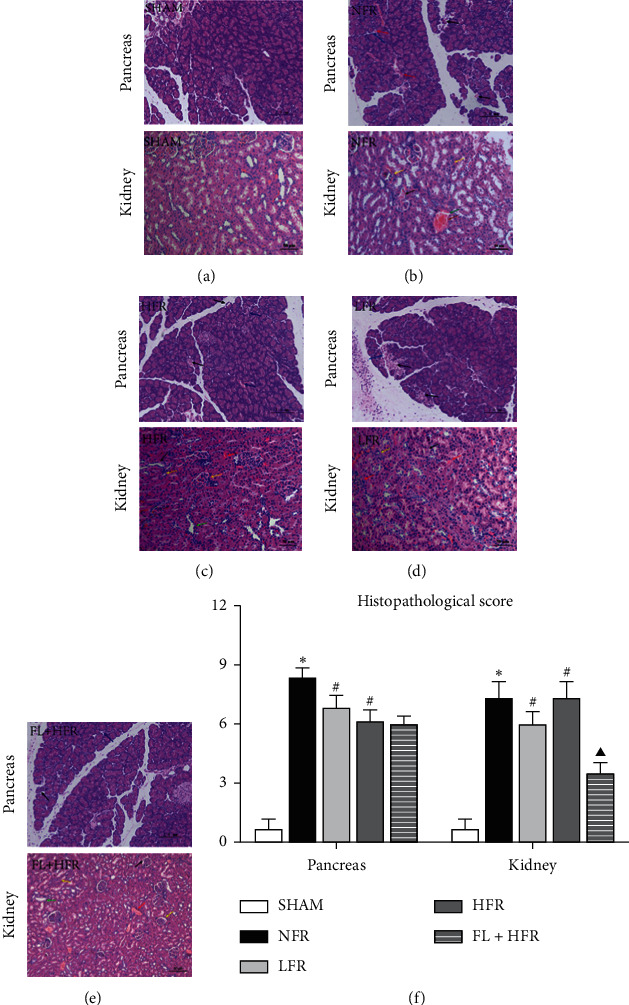
Comparison of pancreatic and renal histopathologies. SHAM: sham; NFR: nonfluid resuscitation; LFR: low-volume fluid resuscitation; HFR: high-volume fluid resuscitation; FL + HFR: *Poria cocos* combined with high-volume fluid resuscitation. Morphological changes of organs including inflammatory infiltration (blue arrow hemorrhage or hyperemia (red arrow), necrosis (black arrow), glomerular atrophy or renal tubular stenosis (yellow arrow), and partial tubular dilation (green arrow) were observed in rats (a–e). The morphological changes of organs in histopathological score (f). ^∗, #, ▼,▲^Compared with the SHAM, NFR, LFR, and HFR groups, respectively, *p* < 0.05.

*(1) Effect of Inflammatory Response of SAP on Organ Function*. The SAP model had obvious intravascular volume depletion, inflammatory exudation, and functional changes of the lung, kidney, and heart. Compared with the sham group (SHAM), the AP model (NFR) had observed elevated white blood cell, hematocrit, oliguria, lactate, and ascites presenting enhanced inflammatory injury, intravascular volume depletion, tissue perfusion dysfunction, and exudative effusion. Increased blood urea nitrogen and creatinine and decreased pH, partial pressure of oxygen, and arterial oxygen saturation were observed in the NFR group presenting the kidney and lung injuries (Figure 3 and Table 1). The mean arterial pressure and heart rate after modeling or laparotomy in the NFR group were higher than those in SHAM (Figure 4). In the NFR group, the morphological changes of the pancreas and kidney in inflammatory infiltration, edema, hemorrhage, and necrosis were obvious, and the pathological scores of the pancreas and kidney were also significant higher than those in the SHAM (Figure 5 and Table 1).

*(2) Effect of Low- and High-Volume Fluid Resuscitation on Organ Function*. Both low- and high-volume fluid resuscitation alleviated the pathological changes in the pancreas, but low volume was better. Low-volume fluid resuscitation (LFR) decreased the blood urea nitrogen, creatinine, and kidney pathological scores than high-volume fluid resuscitation (HFR).

HFR increased AKI. HFR improved pH, partial pressure of oxygen, lactate, and oliguria than LFR at 10 hours after AP onset, but increased ascites, blood urea nitrogen, creatinine, and kidney pathological scores aggravating exudative effusion and kidney function and pathological injury than LFR (Figure 3 and Table 1). In the comparison of time points in the NFR, LFR, and HFR groups, the mean arterial pressure and heart rate increased to the highest point after the modeling and then declined to the lowest at 3∼6 hours after fluid resuscitation (Figure 4). Obviously, kidney pathological changes in glomerular atrophy and hyperemia, tubular stenosis with partial tubular dilation and tubular collapse, and even structural disorder were observed in the HFR group (Figure 5 and Table 1).

#### 3.2.2. Mechanism of High-Volume Fluid Resuscitation Worsens Organ Injury

**Figure 6:**
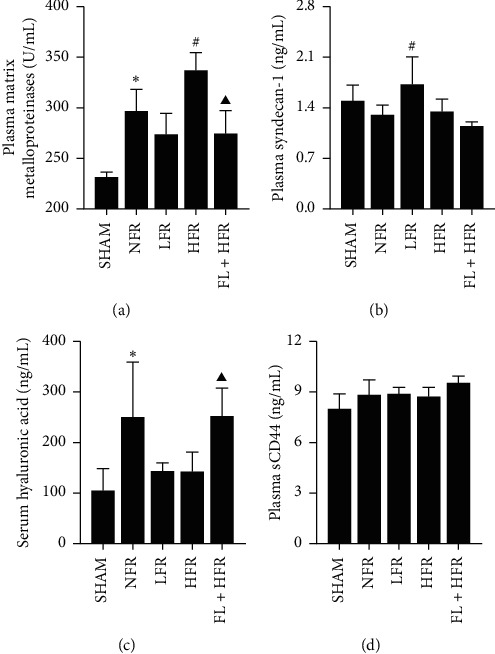
Comparison of glycocalyx composition between five groups. SHAM: sham; NFR: nonfluid resuscitation; LFR: low-volume fluid resuscitation; HFR: high-volume fluid resuscitation; FL + HFR: *Poria cocos* combined with high-volume fluid resuscitation. ^∗, #, ▼, ▲^Compared with the SHAM, NFR, LFR, and HFR groups, respectively, *p* < 0.05.

Endothelial glycocalyx might be involved in organ injury exacerbated by HFR. HFR increased the release of matrix metalloproteinases (MMPs), leading to endothelial glycocalyx degradation and development of AKI (Figure 6 and Table 1). Plasma MMPs and serum hyaluronan of the NFR group significantly increased than those of the SHAM group. MMPs of the HFR group were greater than those in the NFR group (Figure 6 and Table 1). Multiple linear regression found the serum level of glycocalyx components of Syndecan-1, hyaluronan, and soluble CD44 positively correlated with the kidney pathological score. For each unit of Syndecan-1 being increased, the kidney pathological score increased by 1.084 times (Table 2).

#### 3.2.3. Therapeutic Effect and Mechanism of *Poria cocos* Moderated Organ Injury Caused by HFR

*(1) Therapeutic Effect of Poria cocos on Organ Injury Caused by HFR*. The *Poria cocos*-combined high-volume group (FL + HFR) could improve the arterial oxygen saturation, attenuate the inflammatory exudation, and moderate the renal injury aggravated by HFR. Blood urea nitrogen, creatinine, and renal pathological score were lower, and arterial oxygen saturation was higher in the FL + HFR group than that in the HFR group. *Poria cocos* could alleviate the morphological changes of the pancreas and kidney in inflammatory infiltration, edema, hemorrhage, and necrosis than those in the HFR group (Figure 5). Decreased pH and partial pressure of oxygen and increased lactated were observed in the FL + NFR group than those in the HFR group which showed that *Poria cocos* might not improve tissue perfusion and respiratory dysfunction. In the comparison of time points in FL + HFR group, the mean arterial pressure and heart rate increased to the highest point after the modeling and then declined to the lowest at 3∼6 hours after fluid resuscitation and finally to the baseline level at the end of experiment (Figure 4).

*(2) Mechanism of Poria cocos Moderated Organ Injury Caused by HFR*. *Poria cocos* might moderate kidney injury by decreasing MMPs release and reducing endothelial glycocalyx degradation and damage. Plasma MMPs in the FL + NFR group was lower than that in the NFR group. Serum hyaluronan in the FL + NFR group was higher than that in the NFR group. No significant difference of Syndecan-1 and soluble CD44 were found between two groups (Figure 6 and Table 2).

## 4. Discussion

Fluid resuscitation could improve hypovolemia and organ hypoperfusion and reduce extrapancreatic organs injury in the early phase of AP, and early goal-directed therapy (EGDT) was recommended as basic fluid therapy strategy. We found TFR, which is based on the concepts of EGDT, could reduce the fluid volume and velocity and ultimately reduce the AKI secondary to SAP. Meanwhile, increased fluid velocity increased the risk of AKI secondary to SAP. Then, we performed the experimental study to explore the effect of different-volume fluid resuscitation on AKI secondary to SAP and found high-volume exacerbated inflammatory exudation and worsened AKI. Endothelial glycocalyx degradation and vascular endothelial dysfunction might be the cause of high-volume-related AKI in SAP. We found traditional Chinese medicine of *Poria cocos*, which had the functions of promoting water and dampness distribution and metabolism and promoting the spleen to metabolize water and dampness and could moderate kidney injury induced by high-volume through decreasing MMP release and reducing endothelial glycocalyx damage.

SAP patients with AKI accounted from 15.05% to 69.3% in the previous study and 38.7% in our study [4, 5]. Fluid resuscitation is known as the initial management strategy in SAP. The first 48 to 72 h after admission significantly affected prognosis and hospital stay of AP, and the volume of 2500–4000 ml and rate of 250–500 ml/h were recommended as the early fluid resuscitation goals [8, 9]. Therefore, we focused on the first-72 h effect of fluid resuscitation after admission. In our retrospective study, two groups had a 24-hour volume ranged from 2500 to 4000 ml and velocity <250 ml/h. High volume (>4000 ml) as a risk factor increased AKI secondary to SAP and mortality [10, 30]. We found the control group received higher volume (3515.0 vs. 2875.0 ml) and velocity (187.0 vs. 158.0 ml/h) and AKI increased by 1.02 than that of the TFR group. 4 or 8 mL/kg/h fluid resuscitation could alleviate hemoconcentration and acidosis, but higher volume involved arterial hypoxia and ascites which involved organ failure and mortality in AP [31]. We found both low and high volume decreased the hemoconcentration, acidosis, and oliguria and alleviated the pancreas injury, but high volume increased the kidney pathological injury and ascites and decreased the arterial oxygen saturation. Ascites significantly increased the occurrence of organ failure and mortality among AP patients [32]. We found high-volume fluid resuscitation increased the serum creatinine and ascites and decreased the arterial oxygen saturation. *Poria cocos* could reduce abdominal exudation and renal injury and improve arterial oxygen saturation. Previous study shows that *Poria cocos* could improve renal metabolism and reduce kidney injury [33, 34]. We found pathological injury of the kidney aggravated by high volume could be alleviated by *Poria cocos* though promoting distribution and metabolism of fluid. So increased fluid volume, high-volume, particularly, increased the incidence of AKI. SAP patients with AKI possessed higher mortality but our analysis failed to found it [5, 6].

Creatinine is regarded as an index to evaluate the kidney injury according to the Kidney Disease: Improving Global Outcomes and Modified Marshall Scoring System. Creatinine was involved in hemoconcentration and was used to predict the organ failure of AP [35]. We found elevated serum creatinine in the control group which was associated with higher fluid volume compared with that of TFR group. Meanwhile, creatinine of the high-volume group was also significantly higher than that in low-volume group and *Poria cocos* treatment group.

Mean arterial pressure, urine output, heart rate, blood lactic acid, and hematocrit were regarded as indicators of fluid responsiveness measurements [9, 14]. Mean arterial pressure (>65 mmHg) and urine output (>0.5 ml/kg/h) were used to assess the responsiveness of initial intravenous fluid [36]. In our study, there was no significant difference of these indicators except for 24-hour urinary output which was lower in the TFR group compared with that in the control group. After AP modeling, mean arterial pressure, heart rate, blood lactic acid, and hematocrit significantly increased, representing potential injury of the circulatory system, tissue perfusion, and organ. Previous study shows that mean arterial pressure after AP modeling decreased sharply from 131 mmHg to 79 mmHg during eight-hour experiment and began to recover at four hours after fluid resuscitation [37]. Another study found that mean arterial pressure at 12 hours after AP modeling decreased from 115 mmHg to 94 mmHg [38]. We found the mean arterial pressure decreased slightly from 130 mmHg to102.8 mmHg at 8 hours after fluid resuscitation and to 99 mmHg at 12 hours. There was no significant difference between the fluid treatment groups.

Blood lactic acid can predict pancreatic infection and also was suggested as a goal of fluid resuscitation [39, 40]. Blood lactic acid can predict pancreatic infection and even mortality when it is higher than 4 mmol/L [41]. Our retrospective study found three-day mean blood lactic acid was <4 mmol/L and no significant difference between two groups. HFR decreased the serum lactic to improve tissue perfusion. Elevated hematocrit involved hemoconcentration, pancreatic necrosis, organ failure, and mortality, and the target level of 35–44% was suggested [8, 42]. Our retrospective study found the two-group hematocrit level, respectively, declined to 42% and 39%, after three-day fluid resuscitation. Rapid decent of hematocrit involved fluid overload, sepsis, and mortality in AP [11]. We found sharply hematocrit decent in the larger volume group, namely, the control group, which might contribute to AKI. Hematocrit of HFR group was higher than that of the other groups, but there was no statistical difference. These might show that increased volume could not improve circulatory capacity effectively because elevated hematocrit exited still. Meanwhile, hematocrit alone to evaluate the fluid responsiveness might include fluid overload and ineffective hydration.

There were many scoring systems to evaluate the severity and predict the prognosis of AP. Based on the Modified Marshall scoring system, we evaluated the organ damage and dysfunction between two groups [23]. Oxygenation index (PaO_2_/FIO_2_ ratio), systolic blood pressure, and bilirubin were regarded as physiologic measures to assess the organ function [23]. Study reported that the goal-directed fluid group involved a lower mechanical ventilation duration and organ dysfunction than in the control group [12]. We found lower Marshall scores and higher PaO_2_/FIO_2_ ratio at the first 24 h in the TFR group compared with that in the control group. As for use of mechanical ventilation, the TFR group started later with shorter duration than the control group.

Fluid resuscitation could restore the circulatory capacity, but perfusion recovery might be uneven which involved insufficient tissue oxygen supply and microcirculation disorders [43]. Matrix metalloproteinase (MMP) is an important cutter of endothelial glycocalyx causing glycocalyx degradation which involves endothelium and even microcirculation dysfunction [20]. High volume aggravated the glycocalyx degradation, but glycocalyx component degradation varies [44]. The kidney clearance rate of syndecan-1 was higher than that of hyaluronan, but they were cleared completely at 12–15 h after surgery [45, 46]. Plasma syndecan-1 fluctuated greater than hyaluronan, and the elevated urine output increased the kidney clearance rate; therefore, when plasma concentration changed >5–6 times, glycocalyx degradation was considered [46]. We found MMPs of the HFR group was the highest, but the plasma glycocalyx component level was lower, which might result from increased urine-associated enhanced kidney clearance ability. There was no significant difference in glycocalyx component among five groups as well. With the recovery of muscle tension, lymphatic reflux increased and led to the increased reflux of hyaluronan from interstitial fluid, and the plasma hyaluronan concentration would increase significantly after the operation [47]. Additionally, the elution of lymph led to the increased reflux of hyaluronic acid because of the effective hydration of Ringer's solution [48]. *Porio cocos* reduced the release of MMPs and alleviated the glycocalyx damage aggravated by high volume, but *Porio cocos* increased the serum hyaluronan level. *Porio cocos* improved the humoral metabolism and increased humoral fluid exchange between vascular and tissue which probably led to an increase in the serum hyaluronan level.

There were some limitations in our study. This small-sample and retrospective study could not provide strong evidence on the effect of TFR involving lower volume that decreased AKI secondary to SAP. We cannot provide strong evidence on significant differences of glycocalyx degradation among different volume fluid resuscitation.

## 5. Conclusion

In conclusion, TFR associated with lower fluid volume and velocity reduced AKI secondary to SAP. High volume stimulating MMP release increased the endothelial glycocalyx degradation and aggravated AKI. *Poria cocos* reduced the MMP release, relieved the glycocalyx injury, and alleviated the pancreas and kidney injuries aggravated by high fluid volume in SAP. Thus, fluid volume optimization might reduce AKI, and endothelial glycocalyx protection might be a new strategy in the treatment of SAP [49].

## Figures and Tables

**Table 1:** Comparison of organ injury indexes between groups.

	SHAM (*n* = 6)	NFR (*n* = 6)	LFR (*n* = 6)	HFR (*n* = 6)	FL + HFR (*n* = 6)
Hematologic analysis					
AMY (U/L)	992.2 ± 152.4	4618.7 ± 1239^*∗*^	3233.8 ± 1064.6	3292.3 ± 541.6^#^	3437.2 ± 980.8
WBC (10^9^/L)	9.6 ± 1.3	12.2 ± 1.8^*∗*^	9.3 ± 2.8	8.1 ± 0.9^#^	10.6 ± 2.5
HCT (%)	45.3 ± 3.6	51.0 ± 5.1^*∗*^	50.5 ± 2.8	52.0 ± 0.9	47.9 ± 2.5
BUN (mmol/L)	3.0 ± 0.6	12.9 ± 4.6^*∗*^	4.3 ± 0.4^#^	6.4 ± 1.1^#▼^	3.3 ± 0.4^#▼▲^
Cr (*μ*mol/L)	28.7 ± 2.9	44.8 ± 15.4^*∗*^	30.7 ± 2.7^#^	41.5 ± 7.2^▼^	27.0 ± 1.1^#▲^
LAC (mmol/L)	4.1 ± 0.9	5.6 ± 1.2^*∗*^	5.8 ± 1.0	4.0 ± 0.4^#▼^	5.2 ± 0.5^▲^
Ph	7.37 ± 0.1	7.22 ± 0.1^*∗*^	7.27 ± 0.04	7.30 ± 0.1^#^	7.16 ± 0.02^▼▲^
PaO_2_ (mmHg)	92.6 ± 6.9	75.3 ± 2.6^*∗*^	75.9 ± 7.6	122.0 ± 0.7^#^	100.2 ± 3.5^#▲^
SaO_2_ (%)	90.2 ± 3.2	86.3 ± 1.6^*∗*^	86.5 ± 7.0	85.9 ± 1.9	93.6 ± 1.0^#▼▲^
Biological records					
IAC (cm)	16.2 ± 0.8	16.6 ± 0.4	16.7 ± 0.4	16.6 ± 0.5	16.8 ± 0.3
ACI (cm)	0.5 ± 0.3	1.2 ± 0.2^*∗*^	1.3 ± 0.4	1.7 ± 0.4^#^	2.1 ± 0.8^#^
Ascites (mL)	0.6 ± 0.2	5.2 ± 0.3^*∗*^	4.9 ± 2.0	12.8 ± 1.8^#▼^	9.9 ± 2.1^#▼^
Urine output (mL)	2.2 ± 0.7	0.9 ± 0.5^*∗*^	2.2 ± 0.4^#^	3.5 ± 1.7^#^	2.4 ± 1.4^#^
Glycocalyx compositions					
MMPs (U/mL)	231.6 ± 4.7	297.1 ± 21.1^*∗*^	273.8 ± 20.4	337.4 ± 17.3^#^	274.9 ± 22.2^▲^
SCD-1 (ng/mL)	1.5 ± 0.2	1.3 ± 0.1	1.7 ± 0.4	1.4 ± 0.2	1.2 ± 0.1^▼^
sCD44 (ng/mL)	8.0 ± 0.9	8.9 ± 0.9	8.9 ± 0.4	8.7 ± 0.6	9.6 ± 0.4^▲^
HA (ng/mL)	105.9 ± 43	251.2 ± 107.7^*∗*^	143.4 ± 17.2	143.2 ± 38.2	252.6 ± 55.3^▼▲^
Pathological scores					
Pancreas	0.7 ± 0.5	8.3 ± 0.5^*∗*^	6.8 ± 0.6^#^	6.2 ± 0.5^#^	6.0 ± 0.4^#^
Kidney	0.7 ± 0.5	7.3 ± 0.8^*∗*^	6.0 ± 0.6^#^	7.3 ± 0.8	3.5 ± 0.5^#▲^

SHAM: sham; NFR: nonfluid resuscitation; LFR: low-volume fluid resuscitation; HFR: high-volume fluid resuscitation; FL + HFR: *Poria cocos* combined with high-volume fluid resuscitation. AMY: amylase; WBC: white blood cells; HCT: hematocrit; BUN: blood urea nitrogen; Cr: creatinine; LAC: lactate; PaO_2_: partial pressure of oxygen; SaO_2_: arterial oxygen saturation; IAC: initial abdominal circumference; ACI: abdominal circumference increment; MMPs: matrix metalloproteinases; SCD-1: syndecan-1; sCD44: soluble CD44; HA: plasma: hyaluronic acid; ^∗, #, ▼, ▲^Compared with the SHAM, NFR, LFR, and HFR groups, respectively, *p* < 0.05.

**Table 2:** Risk factors of AKI secondary to AP.

	*B*	*t*	*p*	*F*	*P*	Adj. *R*^2^
Constant	−1.557	−4.818	0.04^*∗*^	352.666	0.003^*∗*^	0.995
HA (ng/mL)	0.005	15.758	0.004^*∗*^			
sCD44 (ng/mL)	0.737	29.75	0.001^*∗*^			
SCD-1 (ng/mL)	1.084	8.665	0.013^*∗*^			

AP: acute pancreatits; AKI: acute kidney injury; HA: hyaluronic acid; sCD44: soluble CD44; SCD-1: syndecan-1; Adj: adjusted. ^*∗*^*p* < 0.05.

## Data Availability

All data used to support the results of this study are included within the article and are available from the corresponding author.
